# Selected Serum Biomarkers in Patients with Relapsing-Remitting Multiple Sclerosis—A 3-Year Prospective Pilot Study

**DOI:** 10.3390/medsci13040283

**Published:** 2025-11-25

**Authors:** Przemyslaw Puz, Katarzyna Maciejowska, Daria Gendosz de Carrillo, Malgorzata Janik, Anetta Lasek-Bal

**Affiliations:** 1 Department of Neurology, Faculty of Health Sciences in Katowice, Medical University of Silesia, 40-635 Katowice, Poland; 2 Department of Physiology, Faculty of Medical Sciences in Katowice, Medical University of Silesia in Katowice, 40-752 Katowice, Poland; 3 Department of Histology and Cell Pathology, Faculty of Medical Sciences in Zabrze, Medical University of Silesia in Katowice, 41-808 Zabrze, Poland; 4 Faculty of Science and Technology, Institute of Biomedical Engineering, University of Silesia in Katowice, 41-200 Sosnowiec, Poland

**Keywords:** multiple sclerosis, biomarkers, osteopontin, occludin, neurofilament light chain

## Abstract

Background: The aim of this study was to evaluate the significance of serum concentrations of the inflammatory marker osteopontin, the blood–brain barrier damage marker occludin, and the neurodegeneration marker neurofilament light chain (NFL) in patients with relapsing-remitting multiple sclerosis (RRMS) for predicting disease activity and progression. Methods: This prospective cohort study enrolled 150 patients with RRMS. Initial serum levels of NFL, occludin, and osteopontin were compared between patients who met or did not meet the no evidence of disease activity (NEDA) criteria and its components (relapses, magnetic resonance imaging activity, and Expanded Disability Status Scale progression) within 36 months of observation. Independent factors affecting study outcomes at month 36 were identified from baseline data, including age, gender, initial prognostic profile, and serum levels of NFL, occludin, and osteopontin, as well as treatment type. Results: We found lower osteopontin concentrations in patients receiving highly effective treatment compared with those receiving platform therapies (13.64 ± 5.41 ng/mL, CI 11.75–15.53 vs. 17.33 ± 8.00 ng/mL, CI 15.66–18.61; *p* = 0.03). There was a significant correlation between NFL levels and patient age (Spearman’s rho = 0.3045, *p* = 0.0002) and between NFL levels and disease duration (Spearman’s rho = 0.1945, *p* = 0.02). NEDA during the 3-year observation period was achieved by 58 (38.67%) patients. Patients with NEDA showed significantly lower serum concentrations of occludin, NFL, and osteopontin than those without NEDA. Conclusions: Serum levels of NFL, osteopontin, and occludin may serve as biomarkers of disease activity in patients with RRMS. The clinical relevance of these biomarkers should be confirmed through repeated serum marker assessments in MS patients and validation studies involving larger sample sizes.

## 1. Introduction

Multiple sclerosis (MS) is a chronic inflammatory-demyelinating disease of the central nervous system (CNS) [1].

MS typically begins at a young age and, due to its potentially progressive nature, carries a risk of disability. It is estimated that approximately 3 million people worldwide are affected by MS [2]. The majority of patients (about 85%) have the relapsing-remitting form of the disease. The main tool used to establish the diagnosis and monitor disease progression is brain magnetic resonance imaging (MRI). However, MRI combined with clinical evaluation does not fully assess patients with MS. In recent years, numerous studies have explored the use of laboratory biomarkers to determine the prognosis of patients with MS. Identifying such a biomarker (or group of biomarkers) remains one of the key objectives of MS research [3,4,5,6].

The literature provides information on laboratory biomarker assessment in MS patients. Most findings are based on biomarker determinations in cerebrospinal fluid (CSF). However, obtaining CSF is an invasive and burdensome procedure. Therefore, researchers are seeking biomarkers measurable in blood serum, because this test is more convenient and accessible [5,6]. The localization of disease activity within the CNS, along with the presence of the blood–brain barrier, limits the potential for identifying an MS-specific serum biomarker that accurately reflects ongoing processes within the CNS. The results of biomarker studies remain inconclusive, and no biomarker has yet achieved the status of a marker used in routine clinical practice [5,6,7].

Recent positive reports on the measurement of serum neurofilament light chains (NFL) have encouraged further investigation in this area.

The aim of this study is to evaluate the significance of the concentrations of selected biomarkers—an inflammatory marker (osteopontin), a marker of blood–brain barrier damage (occludin), and a marker of neurodegeneration (NFL)—in the serum of patients with the relapsing-remitting form of MS, to predict disease activity and disability progression. The study hypothesis is that serum concentrations of the selected biomarkers are significantly higher in patients with active disease and progression compared with those with inactive disease.

## 2. Materials and Methods

This was a prospective cohort study that enrolled 150 patients diagnosed with relapsing-remitting multiple sclerosis (RRMS). Data were collected from patients treated at a single clinical center between January 2020 and December 2024. We included patients with a diagnosis of RRMS according to current McDonald criteria who were receiving disease-modifying treatment (DMT). Initially, 160 patients were included in the study. During the 3-year follow-up period, 10 patients were lost to follow-up due to discontinuation of treatment (3 patients) or transfer to another treatment center (7 patients).

The study was conducted in accordance with the Declaration of Helsinki and was approved by Ethics Committee of Medical University of Silesia (no KWN/0022/KB1/67/I/19; date of approval: 3 December 2019). Informed consent was obtained from all subjects involved in the study.

Inclusion criteria:

- Age 20–55 years
- Diagnosis of MS according to current McDonald criteria
- Expanded Disability Status Scale (EDSS) score below 5.0
- DMT continued for at least 6 months before the first visit and blood sample collection

Exclusion criteria:

1. Inability to provide informed consent to participate in the research.
2. Recent infection (diagnosed clinically and confirmed with basic laboratory tests)
3. Active relapse at enrollment
4. Acute or chronic renal failure
5. Known liver disease or elevated liver function markers (≥2 times the upper limit of normal)
6. Neurologic disease other than MS-related symptoms
7. Cancer
8. Alcohol abuse
9. Hematologic disease
10. Connective tissue disease
11. Chronic inflammatory bowel disease
12. Pancreatitis
13. Hormonal disorders (hypothyroidism, hyperthyroidism, gonadal hormone disorders)
14. Use of steroids within one month prior to enrollment

In all patients, the following data were assessed:

Enrollment assessment:

- Demographics (age, gender), disease duration, treatment duration, type of therapy. Natalizumab, oral cladribine, antiCD20 agents, and S1P modulators were classified as high-efficacy treatments (HET), whereas interferons, dimethyl fumarate, glatiramer acetate, and teriflunomide were classified as moderate-efficacy (platform) therapies.
- Clinical status (EDSS) and clinical activity (number of relapses in the past year)
- Initial laboratory testing of biomarkers (serum NFL, occludin, osteopontin)
- MRI assessment

Based on the initial assessment, patients were divided into two groups: 1. favorable prognostic profile, and 2. unfavorable prognostic profile.

Definition of unfavorable prognostic profile: any four of the following: male sex, age > 45 years, presence of pyramidal signs, presence of cerebellar or spinal symptoms, bladder or bowel dysfunction, more than one relapse in the past year, more than 10 T2 lesions on the last MRI, or more than one gadolinium-enhancing (Gd+) lesion.

Follow-up assessment (visits at months 12, 24, 36):

During each visit, participants were assessed clinically for relapses in the past 12 months and for clinical status using the EDSS.

- MRI was performed at months 12, 24, and 36 using standard sequences (FLAIR, T2, T1, T1 + C) in accordance with the protocol of the Polish Medical Radiology Society.
- Disease activity was defined as the occurrence of at least one clinical relapse, confirmed disability progression (CDP), or MRI activity during each year of DMT. CDP was defined as an increase of at least 1.5 points in EDSS from a baseline score of 0, or an increase of 1 point from a baseline score of 1.0–4.5, sustained at two or more consecutive visits separated by at least 180 days. If a patient reached an EDSS score of 5.0 during the observation period, an increase of 0.5 points was considered progression. A relapse was defined as a clinical episode lasting at least 24 h in the absence of fever, infection, or acute concurrent illness, preceded by a 30-day relapse-free period. MRI activity was defined by the presence of at least one new or enlarged T2 lesion and/or at least one Gd+ lesion.

Patients with relapses and/or CDP and/or MRI activity were classified as having evidence of disease activity (EDA), whereas patients without relapses, CDP, or MRI activity were classified as having no evidence of disease activity (NEDA).

Comparisons

Initial serum levels of NFL, occludin, and osteopontin were compared between patients who met or did not meet NEDA and its components (relapses, MRI activity, EDSS progression) during the 36-month observation period.

Laboratory tests

Fasting blood samples were collected to assess serum biomarkers (NFL, occludin, osteopontin). Blood was allowed to clot, then centrifuged at 3500 rpm for 15 min at room temperature. Serum samples were immediately frozen and stored until analysis. Biomarker levels were determined using ELISA immunoenzymatic assays in duplicate. All measurements were performed with the automated microplate reader ELISA PIOWAY.

Serum concentrations of NFL, occludin and osteopontin were assessed only once at study entry, prior to the initiation of the prospective follow-up.

Statistical analysis

A significance level of α = 0.05 was used to control for a 5% Type I error rate. Multiple testing was addressed using the Benjamini–Hochberg false discovery rate (FDR) procedure. For all subgroup comparisons, raw *p*-values were adjusted for multiple testing and FDR-corrected *p*-values are reported. Descriptive statistics summarized the data: continuous variables were reported as mean (with standard deviation) or median, while categorical variables were described as frequencies (n) and percentages.

The *t*-test or Wilcoxon rank-sum test was used to compare continuous variables between two independent groups, Pearson’s chi-square test or Fisher’s exact test was used for categorical variables, depending on sample size. Spearmann’s test was used to assess correlations between variables.

Multivariate logistic regression models were created to identify whether serum NFL, occludin, osteopontin levels were the factors affecting the study outcomes: NEDA and its compounds (relapses, MRI activity, EDSS progression) at month 36. All multivariable models were adjusted for age, sex, disease duration, treatment class (HET vs. platform therapies), and baseline prognostic profile to account for potential confounding effects.

## 3. Results

### 3.1. Patient Characteristics

Initial patient characteristics are presented in Table 1.

There were no significant differences in serum concentrations of occludin, NFL and osteopontin between men and women and or the group of patients with an unfavorable prognostic profile compared to those with a favorable prognostic profile.

We found lower osteopontin concentrations in patients receiving HET compared with those receiving platform therapies (13.64 ± 5.41, CI 11.75–15.53 vs. 17.33 ± 8.00, CI 15.66–18.61; *p* = 0.028). No statistically significant differences in occludin and NFL serum concentrations according to HET use.

There was a statistically significant correlation of NFL levels with patient age (Spearman’s rho 0.3045, *p* = 0.0002) and disease duration (Spearman’s rho 0.1945, *p* = 0.0172). No significant correlation was observed between occludin or osteopontin levels and patient age or disease duration.

### 3.2. Predicting Disease Activity and Disability Progression

During the 3-year observation period, CDP occurred in 49 patients (32.67%), clinical relapses in 45 patients (30%), new or enlarged T2 lesions on MRI in 65 patients (43.33%), and Gd+ lesions in 30 patients (20%). NEDA status over 3 years was achieved in 58 patients (38.67%).

Patients with NEDA had significantly lower serum concentrations of occludin, NFL, and osteopontin compared with patients without NEDA (Table 2).

Serum osteopontin concentrations were significantly higher in patients with EDSS progression compared to those without EDSS progression and in patients with MRI activity compared to those without MRI activity. No significant difference in osteopontin levels was observed between patients with and without relapses. NFL serum levels were significantly higher in patients with relapses than in patients without relapses. There was no significant difference in NFL and occludin levels between patients with and without EDSS progression and between patients with and without new MRI Gd+ lesions. Occludin serum concentrations were significantly higher in patients with new (or enlarged) T2 lesions on MRI (Table 2 and Table 3).

In multivariable logistic regression analysis including serum biomarkers, age, sex, disease duration, treatment type and unfavorable initial prognostic profile serum concentrations of studied biomarkers were the independent predictors of EDA achievement (Table 4). Occludin levels were associated with the occurrence of new (or enlarged) MRI T2 lesions. NFL levels were associated with EDSS progression, clinical relapses and the occurrence of new (or enlarged) MRI T2 lesions. Osteopontin levels were associated with EDSS progression, the occurrence of new (or enlarged) MRI T2 lesions and the presence of active MRI (Gd+) lesions (Table 5 and Table 6).

## 4. Discussion

Significantly lower serum concentrations of occludin, NFL, and osteopontin in patients with NEDA as compared to patients with those without NEDA are the main findings of our study.

NFL serum levels are a recognized marker of clinical and radiological disease activity, confirmed in numerous studies; however, due to low specificity and complex measurement methodology, they have not been implemented into common routine clinical practice [8,9]. Therefore, other biomarkers of disease activity in MS patients are being investigated. Laboratory biomarkers are being studied to aid diagnostic and prognostic assessment, determine the risk of MS onset and its natural course or activity, and evaluate treatment response [5,6,7,8,9,10,11].

The presence of the blood–brain barrier makes it difficult to obtain a serum marker specific to MS that would allow for reliable assessment of disease progression and activity [10,11,12]. The results of studies on serum biomarkers in multiple sclerosis are inconclusive, and there is still a need to identify a marker that could be useful in routine clinical practice [10,11,12,13,14].

In our study, we confirmed the importance of NFL for predicting clinical disease activity. We also found that higher osteopontin concentrations were associated with increased risk of radiological disease activity and progression of patients’ disability, and higher occludin concentrations with increased risk of radiological disease activity.

The associations observed for osteopontin and occludin may reflect a biological interplay between inflammatory activation and blood–brain barrier integrity. Osteopontin upregulation is known to induce matrix metalloproteinases—particularly MMP-2 and MMP-9—which degrade tight-junction proteins such as occludin. This degradation contributes to increased blood–brain barrier permeability and facilitates leukocyte migration into the CNS. Thus, elevated osteopontin levels could drive endothelial damage through MMP-mediated tight-junction disruption, providing a biologically plausible link between systemic inflammation and blood–brain barrier dysfunction. Such a mechanism may help explain why osteopontin and occludin show concordant changes in patients with active disease and suggests that these biomarkers capture complementary dimensions of MS-related neuroinflammation and barrier disturbance [15,16].

In multivariable logistic regression analysis including serum biomarkers, age, sex, disease duration, treatment and unfavorable prognostic profile of initial patients characteristics, we found associations of occludin levels with EDSS progression and the occurrence of new (or enlarged) MRI T2 lesions, NFL levels with EDSS progression, clinical relapses and the occurrence of new (or enlarged) MRI T2 lesions as well as osteopontin levels with EDSS progression, the occurrence of new (or enlarged) MRI T2 lesions and the presence of active MRI (Gd+) lesions.

Our results are consistent with and extend previous studies on multiple sclerosis biomarkers. Previous studies have found higher NFL, occludin and osteopontin concentrations in MS patients compared to healthy controls [17,18,19,20,21]. Occludin and osteopontin levels were associated with disease activity and progression. In the study by Camara-Lemarroy et al., patients with active Gd+ MRI lesions had higher occludin levels compared to patients without active MRI lesions [17]. Disanto et al. demonstrated that elevated serum NFL concentrations robustly correlate with MRI activity, relapse occurrence, and disability progression in large prospective MS cohorts, supporting NFL as a sensitive marker of ongoing axonal injury [18]. Consistent with those observations, we found that higher baseline NFL serum concentrations were associated with both clinical and radiological disease activity during follow-up. Similarly, our results regarding osteopontin agree with Stampanoni et al., who reported increased osteopontin levels in MS patients with active disease, implicating osteopontin in pathways linking immune activation to CNS injury [20]. Taken together, our data are in line with prior evidence and reinforce the relevance of NFL, occludin and osteopontin as complementary biomarkers capturing distinct aspects of MS-related neuroinflammation, neuroaxonal damage, and blood–brain barrier dysfunction.

The significance of these markers for assessing the risk of disease activity in multivariate analysis supports their potential usefulness in clinical practice. Identifying predictors of disease activity, progression, and response to DMT is a current focus of MS research [22,23]. Widely recognized unfavorable prognostic predictors now allow early selection of appropriate therapy [24,25]. The ability to use one or more serum biomarkers could facilitate clinical decision-making in diagnosis and treatment planning. Given the limited efficacy and side effects of current therapies, additional biomarker information could help clinicians optimize treatment selection and the timing of escalation at appropriate disease stages.

### 4.1. Strengths of the Study

Our study is based on real-world clinical evidence and includes three years of prospective follow-up, which strengthens its relevance for clinical practice. The use of serum as the source for biomarkers of disease activity, rather than CSF, aligns with current research trends in the MS field.

### 4.2. Study Limitations

The present study has several limitations. First, the cohort was recruited from a single clinical center and included patients treated with eight different disease-modifying therapies. This treatment heterogeneity reflects real-world clinical practice but may introduce variability that reduces statistical power to detect subgroup-specific effects. Treatment choice itself is influenced by the individual prognostic profile, which may further confound the relationship between serum biomarkers and clinical or radiological outcomes. Although treatment type (highly effective therapy vs. platform therapy) was included as a covariate in the multivariable regression models, residual confounding cannot be excluded. Second, the sample size, although adequate for the main analyses, limits the ability to perform more detailed stratification by specific treatment categories. Third, determinations of the biomarkers studied were performed once, at the beginning of follow-up. Performing subsequent determinations, at specific time points, would allow us to assess the dynamics of changes in the biomarkers studied, which would undoubtedly allow us to better understand their relationship to clinical and radiological activity of the disease. Lastly, as this was an observational, single-center study, the findings should be validated in larger, multicenter cohorts with standardized biomarker collection protocols. The exploratory nature of this study and the single baseline measurement of biomarkers did not allow for assessment of diagnostic performance or determination of optimal cut-off values. Accordingly, ROC curve analyses were not undertaken, as they would lack statistical robustness and external validity in this cohort. Future studies incorporating repeated biomarker sampling, larger sample sizes, and external validation will be necessary to evaluate diagnostic accuracy and establish clinically meaningful thresholds.

## 5. Conclusions

Serum concentrations of NFL, osteopontin, and occludin may serve as independent biomarkers of disease activity in patients with RRMS. The clinical relevance of these biomarkers requires confirmation through repeated measurements of serum marker levels in MS patients, as well as validation in studies with larger sample sizes.

## Figures and Tables

**Table 1:** Patient characteristics.

Gender (F/M)	102/48 (68/32%)
Age (mean ± SD) (years)	40.53 ± 8.03 (22–58)
Disease duration (mean ± SD) (years)	8.41 ± 4.39 (1–32)
EDSS (median, range)	2.0 (0–6.0)
Patients with relapses in last year (n, %)	24 (16%)
EDSS change last year, (n, %)	21 (14%)
MRI new T2 lesions last year (n, %)	31 (20.67%)
MRI Gd+ last year (n, %)	14 (14%)
NEDA last year, (n, %)	101 (67.33%)
Unfavorable prognostic profile (n, %)	64 (42.67%)
Treatment	n (%)
DMF	66 (44%)
GA	14 (9.3%)
INF	24 (16%)
TFL	12 (8%)
Natalizumab	17 (11.3%)
Fingolimod	12 (8%)
Cladribine	2 (1.3%)
Ocrelizumab	3 (2%)

**Table 2:** Biomarkers in patients with clinical disease activity.

	EDSS Progression		Relapses		NEDA	
	Yes	No	Yes	No	Yes	No
N	47	101	45	104	56	89
Occludin [ng/mL]						
Mean	7.42	6.95	7.58	6.90	6.47	7.50
SD	2.09	1.80	2.06	1.81	1.61	1.98
95% CI	6.82–8.02	6.59–7.31	6.96–8.20	6.55–7.25	6.05–6.89	7.09–7.91
*p* ^1^	0.218		0.067		0.0025	
NFL [ng/mL]						
Mean	265.61	193.92	309.40	177.89	156.33	255.80
SD	230.82	149.09	228.99	142.02	97.86	211.01
95% CI	199.31–331.91	164.49–223.36	240.60–379.19	150.40–205.47	130.6–182.06	212.1–299.50
*p* ^2^	0.3		0.004		0.045	
Osteopontin [ng/mL]						
Mean	22.04	13.58	17.36	15.90	12.57	18.72
SD	9.15	4.79	8.52	7.21	3.82	8.44
95% CI	19.41–22.67	12.63–14.52	14.80–19.92	14.51–17.30	11.56–13.57	16.97–20.47
*p* ^2^	0.0003		0.47		0.0003	

^1^—*t*-test, ^2^—Wilcoxon rank-sum test, all *p*-values presented are FDR-adjusted using the Benjamini–Hochberg method.

**Table 3:** Biomarkers in patients with radiological activity.

	New (or Enlarged) MRI T2 Lesions		New MRI Gd+ Lesions	
	Yes	No	Yes	No
N	61	81	26	116
Occludin [ng/mL]				
Mean	7.83	6.55	7.33	7.05
SD	1.88	1.74	1.84	1.93
95% CI	7.36–8.29	6.17–6.92	6.64–8.01	6.70–7.40
*p* ^1^	0.0003		0.48	
NFL [ng/mL]				
Mean	271.65	175.81	247.21	209.87
SD	224.69	128.18	209.08	175.1
95% CI	215.98–327.33	148.15–203.45	169.14–325.29	178.22–241.52
*p* ^2^	0.067		0.38	
Osteopontin [ng/mL]				
Mean	19.72	13.76	24.17	14.39
SD	8.97	5.12	8.92	5.84
95% CI	17.50–21.94	12.65–14.86	20.83–27.50	13.33–15.44
*p* ^2^	0.0003		0.0003	

^1^—*t*-test, ^2^—Wilcoxon rank sum test, all *p*-values presented are FDR-adjusted using the Benjamini–Hochberg method.

**Table 4:** Multivariate regression analysis for factors associated with evidence of disease activity.

	EDA		
	OR	*p*	95% CI
Occludin	1.40	0.009	1.08–1.79
NFL	1.005	0.01	1.002–1.008
Osteopontin	1.22	<0.001	1.12–1.33
Unfavorable prognostic profile	1.12	0.81	0.45–2.76
Sex (Male)	1.26	0.6	0.52–3.04
Age	0.97	0.37	0.91–1.03
Disease duration	0.99	0.8	0.88–1.11
No HET	1.3	0.59	0.5–3.37

**Table 5:** Multivariate regression analysis for factors associated with clinical disease activity.

	EDSS Progression			Relapses		
	OR	*p*	95% CI	OR	*p*	95% CI
Occludin	1.04	0.76	0.82–1.32	1.13	0.28	0.91–1.40
NFL	1.003	0.016	1.0005–1.006	1.004	0.001	1.001–1.005
Osteopontin	1.23	<0.001	1.14–1.32	1.03	0.25	0.98–1.08
Unfavorable prognostic profile	0.54	0.25	0.19–1.54	0.84	0.69	0.35–2.02
Sex (Male)	1.58	0.32	0.64–3.86	0.66	0.33	0.28–1.52
Age	0.98	0.67	0.92–1.05	1.001	0.96	0.94–1.06
Disease duration	1.06	0.37	0.93–1.20	1.01	0.77	0.91–1.13
No HET	2.20	0.13	0.80–6.03	1.42	0.45	0.57–3.54

**Table 6:** Multivariate regression analysis for factors associated with radiological disease activity.

	New (or Enlarged) MRI T2 Lesions			New MRI Gd+ Lesions		
	OR	*p*	95% CI	OR	*p*	95% CI
Occludin	1.56	0.001	1.20–2.02	1.05	0.75	0.78–1.42
NFL	1.004	0.008	1.001–1.006	1.001	0.51	0.99–1.004
Osteopontin	1.15	<0.001	1.08–1.23	1.20	<0.001	1.12–1.29
Unfavorable prognostic profile	1.59	0.31	0.64–3.94	3.01	0.07	0.93–9.77
Sex (Male)	1.64	0.24	0.71–3.76	0.87	0.79	0.3–2.51
Age	0.98	0.73	0.93–1.05	0.97	0.38	0.89–1.04
Disease duration	0.98	0.76	0.88–1.1	0.93	0.33	0.80–1.08
No HET	0.74	0.54	0.28–1.95	0.92	0.89	0.26–3.25

## Data Availability

The data presented in this study are available on request from the corresponding author. The data are not publicly available due to potentially sensitive and personal information. De-identified or aggregated datasets can be shared with qualified researchers upon reasonable request.

## Abbreviations

The following abbreviations are used in this manuscript:

MS	Multiple sclerosis
CNS	Central nervous system
MRI	Magnetic resonance imaging
CSF	cerebrospinal fluid
NFL	neurofilament light chains
RRMS	relapsing-remitting multiple sclerosis
EDSS	Expanded Disability Status Scale
HET	high effective treatment
DMF	dimethyl fumarate
GA	glatiramer acetate
CDP	confirmed disability progression
EDA	evidence for disease activity
NEDA	evidence for disease activity
